# Does Long-Term Care Policy Enable or Limit Volunteers’ Roles in Enhancing Resident Quality of Life?

**DOI:** 10.1093/geroni/igab046.1429

**Published:** 2021-12-17

**Authors:** Emily Hubley, Mary Jean Hande

**Affiliations:** Mount Saint Vincent University, Halifax, Nova Scotia, Canada

## Abstract

This paper examines how volunteer roles are represented in Canadian long term care (LTC) policy in four Canadian jurisdictions, attending to how these regulated roles might impact resident quality of life. Overall, we found that policies define volunteer roles narrowly, which may limit residents’ quality of life. This happens through (1) omitting volunteers from most regulatory policy, (2) likening volunteers to supplementary staff rather than caregivers with unique roles, and (3) over-emphasizing residents’ safety, security and order. We offer insights into promising provincial policy directions for LTC volunteers, yet we caution against further regulating volunteers. Instead, we argue, addressing the cultural, social and structural changes required for volunteers to enhance LTC residents’ quality of life effectively.

